# Substance use disorders in prisoners: an updated systematic review and meta‐regression analysis in recently incarcerated men and women

**DOI:** 10.1111/add.13877

**Published:** 2017-06-28

**Authors:** Seena Fazel, Isabel A. Yoon, Adrian J. Hayes

**Affiliations:** ^1^ Department of Psychiatry University of Oxford, Warneford Hospital Oxford UK

## Abstract

**Aims:**

The aims were to (1) estimate the prevalence of alcohol and drug use disorders in prisoners on reception to prison and (2) estimate and test sources of between study heterogeneity.

**Methods:**

Studies reporting the 12‐month prevalence of alcohol and drug use disorders in prisoners on reception to prison from 1 January 1966 to 11 August 2015 were identified from seven bibliographic indexes. Primary studies involving clinical interviews or validated instruments leading to DSM or ICD diagnoses were included; self‐report surveys and investigations that assessed individuals more than 3 months after arrival to prison were not. Random‐effects meta‐analysis and subgroup and meta‐regression analyses were conducted. Preferred Reporting Items for Systematic Reviews and Meta‐Analyses (PRISMA) guidelines were followed.

**Results:**

In total, 24 studies with a total of 18 388 prisoners across 10 countries were identified. The random‐effects pooled prevalence estimate of alcohol use disorder was 24% [95% confidence interval (CI) = 21–27], with very high heterogeneity (*I* ^2^ = 94%). These ranged from 16 to 51% in male and 10–30% in female prisoners. For drug use disorders, there was evidence of heterogeneity by sex, and the pooled prevalence estimate in male prisoners was 30% (95% CI = 22–38; *I* ^2^ = 98%; 13 studies; range 10–61%) and, in female prisoners, was 51% (95% CI = 43–58; *I* ^2^ = 95%; 10 studies; range 30–69%). On meta‐regression, sources of heterogeneity included higher prevalence of drug use disorders in women, increasing rates of drug use disorders in recent decades, and participation rate.

**Conclusions:**

Substance use disorders are highly prevalent in prisoners. Approximately a quarter of newly incarcerated prisoners of both sexes had an alcohol use disorder, and the prevalence of a drug use disorder was at least as high in men, and higher in women.

## Introduction

Prisons around the world detain large numbers of individuals with substance use problems, which increase the risk of mortality after prison release 1, 2, 3 and repeat offending 4, 5. In addition, alcohol use disorders (AUD) are associated with suicide inside prison 6 and of perpetrating violence and being victimized inside custody 7, 8.

The treatment gap for substance use disorders (SUD) inside prison has been reported in many studies 9, 10. Estimates of the prevalence of these disorders in prisoners can assist in planning service provision effectively, targeting scarce resources and developing and evaluating initiatives to reduce the gap between health needs and interventions. A previous systematic review reported ranges for drug abuse and dependence of 10–48% in men and 30–60% in women on reception or arrival to prison. For alcohol abuse and dependence, ranges of 18–30% for men and 10–24% for women were reported 11. There were very high rates of heterogeneity between these included studies (with *I* ^2^ values of more than 80%), which were investigated in subgroup analyses. Lower prevalences were associated with studies where psychiatrists acted as interviewers and higher prevalences for drug use disorders in remand prisoners. However, this review is now dated, with its search for primary studies ending in 2004, and a number of relevant investigations have been published subsequently. In addition, subgroup analyses were the limited number of primary studies by sex, and an updated review will allow for further investigation of sources of between‐study variation.

The aim of the current paper is to provide an update of prevalence estimates of alcohol and drug use disorders in prisoners and estimate sources of between‐study heterogeneity. As part of this, we have used the term ‘substance use disorder’, which does not distinguish between ‘abuse’ and ‘dependence’. In this update, we have also conducted meta‐analyses to report pooled prevalence estimates and meta‐regression to examine sources of variation between included studies.

## Methods

### Search strategy

We identified surveys of alcohol and drug use disorder in general prison populations (defined as remand/detainee and/or sentenced prisoners who are sampled from the whole population of a correctional institution) published between January 1966 and August 2015. For the period January 1966 and January 2004, methods have been described in a previous systematic review conducted by one of the authors (S.F.) 11. For this update, we searched the following databases from 1 January 2004 to 11 August 2015: PsycINFO, MEDLINE, Global Health, PubMed, CINAHL, National Criminal Justice Reference Service and EMBASE. We used a combination of search terms relating to substance use disorder (i.e. substance*, alcohol, drug*, misuse, dependen*, abuse) and prisoners (i.e. inmate*, sentenced, remand, detainee*, felon*, prison*, incarcerat*), which are same search terms used in the previous review except for the addition of ‘incarcerat*’. Additional targeted searches covered relevant reference lists, and non‐English papers were translated. We corresponded with authors to clarify data when necessary. We followed the Preferred Reporting Items for Systematic Reviews and Meta‐Analyses (PRISMA) guidelines 12 (Supporting information, Appendix S1) and registered the protocol for this review with PROSPERO (registration code CRD42016036416) 13.

### Study eligibility

Inclusion criteria were studies: (a) reporting diagnoses of substance use disorder (i.e*.* substance abuse and/or dependence) based on clinical examination or by interviews using validated diagnostic instruments [based on DSM (versions III to IV‐R; codes 303.90, 304.00–90, 305.00–90, excluding nicotine‐related disorders) and ICD versions 9 and 10 (ICD‐9: 303–305; ICD‐10 codes: F10–19.1‐2 except F17)]; (b) with diagnoses based on the previous 12 months from the time when participants were interviewed/examined; and (c) that sampled the general prison population within 3 months of arrival to prison. We excluded studies that selected subgroups for interview (e.g. prisoners referred for treatment, specific categories of offenders), as the aim was to provide a prevalence estimate for the whole prison population 14, 15, 16. After correspondence with authors, if studies reported combined prevalence for alcohol and drug 17, 18 or combined male and female prevalence, these were excluded 19, as we aimed to report estimates separately by sex and by drug and alcohol use disorder. Studies that reported specific drugs 20, 21, self‐screening measures 22, 23 or solely life‐time prevalence were also excluded 24.

Publications in any language were included in the search: studies from low‐ and middle‐income (LMI) countries were reported separately, given high heterogeneity 25, 26. Similarly, studies with juvenile/youth prisoners were analysed separately 27, 28, 29, 30, 31.

### Data extraction and analysis

Two researchers (I.Y. and A.H.) extracted independently information on year of publication, geographical location, total sample, sex, prisoner status (remand/sentenced), average age, method of sampling, sample size, participation rate, type of interviewer, diagnostic instrument, diagnostic criteria (ICD versus DSM) and number diagnosed with substance use disorders. If older studies reported dependence prevalence, this was prioritized over abuse, as we considered that these had higher diagnostic validity 32, 33 (except when only combined prevalence for abuse and dependence was available). Eligible studies were assessed for quality using the JBI Critical Appraisal Checklist for Studies Reporting Prevalence Data, which uses nine criteria including sample size, sampling, sample description, appropriate statistical analysis and response rates (Supporting information, Appendix S2) 34.

We conducted a random‐effects analysis, which assigns similar weights to all studies included in the meta‐analysis regardless of sample size 35. If there were high levels of overall heterogeneity (*I* ^2^ > 75%), we also reported estimate ranges as an alternative. Meta‐regression analysis was performed to examine sources of between‐study heterogeneity on a range of study pre‐specified characteristics [i.e*.* sex, age, publication year, country (United States versus other countries), prisoner status (sentenced versus remand/detainee/unsentenced), participation rate, sample size, diagnostic criteria (ICD versus DSM) and psychiatric interviewer]. Univariable analysis was conducted for both dichotomous and continuous definitions of a variable (e.g. publication year: continuous versus before or after 2000). Multivariable analyses were not conducted due to the limited number of primary studies. If there were fewer than 10 studies that reported an explanatory variable, it was excluded from the meta‐regression 36. Selected continuous variables (study year and proportion sentenced) were converted to dichotomous variables for reporting of pooled prevalence estimates of subgroups. Accordingly, in the meta‐regression, studies that combined both remand and sentenced prisoners were excluded if: (1) prisoner type comprised more than 10% of the total study participants or was unspecified and (2) separate prevalence data were not provided for each type 37, 38, 39, 40, 41, 42.

In addition, pooled prevalence estimates of the subgroups that did not have more than one study in each relevant category were not reported, even if they had significant results on meta‐regression. Further, we conducted subgroup analyses stratified on pre‐specified variables based on our previous review—sex, whether or not the country of origin was United States, remand/detainee versus sentenced prisoner status and whether or not the assessment was conducted by a psychiatrist. We added a new subgroup analysis based on the date of publication (2000, which was approximately the median date). To test for publication bias, funnel plot analysis and Egger's test were conducted on all studies stratified by disorder (i.e. AUD and SUD) and also by sex and disorder 43. Thus, six separate Egger's tests were performed. Studies with juvenile prisoners or LMI countries were not included, as they were clinically heterogeneous and limited in number. The Egger test quantifies bias captured in the funnel plot analysis with linear regression using the value of effect sizes and their precision [standard error (SE)] and assumes that the quality of study conduct is independent of study size 35 All analyses were conducted in Stata (STATA‐IC) version 14 using the following commands: metan (for random‐effects meta‐analysis), metareg (for meta‐regression), metabias (for publication bias analysis) and heterogi (for calculation of confidence intervals for heterogeneity level).

## Results

### Study characteristics

**Figure 1:**
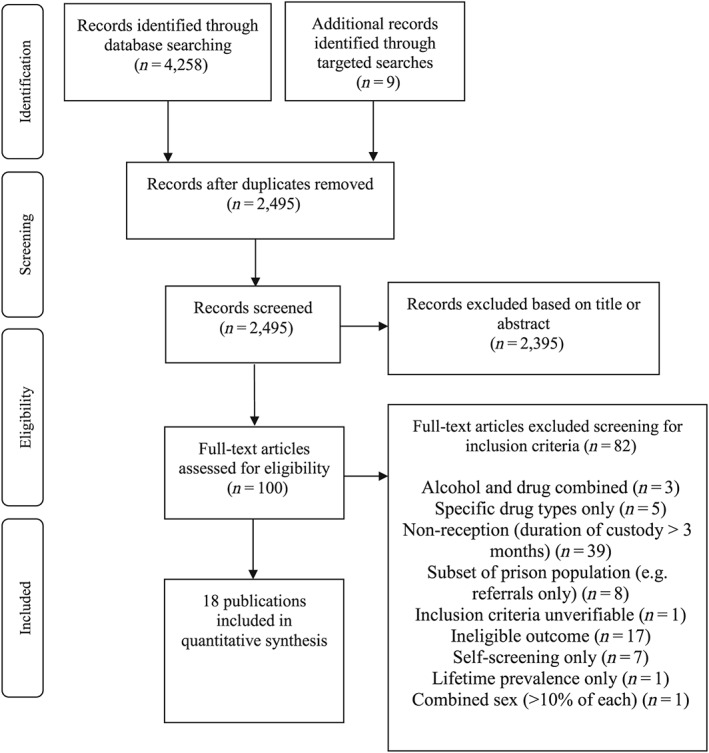
Flow diagram of search strategy for update (2004–15)

We identified 24 publications for the main analysis (Fig. 1), 13 of which were from the previous review 37, 38, 39, 44, 45, 46, 47, 48, 49, 50, 51, 52, 53, and 11 new studies from 2004 40, 41, 42, 54, 55, 56, 57, 58, 59, 60, 61. Two additional studies in LMI countries (Chile 26 and Brazil 25) and five studies on juvenile prisoners (mean age = 16.7 years) were examined separately (Supporting information, Appendix S3) 27, 28, 29, 30, 31.

Studies in the main analysis were from 10 different countries (Australia 40, Austria 61, England 48, France 42, Germany 56, Iceland 60, Ireland 39, 41, 51, 55, the Netherlands 57, New Zealand 37 and United States 38, 44, 45, 46, 47, 49, 50, 52, 54, 59), with 40.5% (7456 prisoners) of the adult combined sample from the United States. Participants were 18 388 prisoners, both sentenced and remand/detainee, 64% of whom were male. The mean age was 30.2 years (range = 17–67 years). Of the 5835 prisoners with criminal history information reported, 924 prisoners (15.8%) were charged or convicted with a violent offence. There were more sentenced (11 065; 60.2%) than remand/detainee/unsentenced prisoners (2975; 16.2%), and 11 investigations included both sentenced and remand prisoners (4348; 23.6%) (‘mixed’ studies) 38, 39, 40, 42, 51, 55, 56, 57. Apart from two studies based on clinical interviews 48, 51, the others involved trained interviewers using validated, structured diagnostic instruments (Table 1 for details). Prevalence of drug use disorder were based on all drugs excluding alcohol and tobacco (i.e. cannabis, opioids, cocaine, amphetamine, hallucinogens, inhalants, other stimulants and tranquillizers). The individual prevalence estimates of substance use disorders are summarized in Table 2. In terms of quality of the included studies, we determined that nine of 24 studies were of high quality, as they met all nine criteria in the quality checklist, including a sufficient sample size (> 250), low refusal rate (< 20%) and detailed description of study subjects and setting 38, 41, 42, 44, 46, 49, 53, 54, 59 (see Supporting information, Appendix S2 for full criteria).

**Table 1:** Study characteristics of newly included studies of substance use disorder in prisoners on arrival into custody (by study year).

Study	Country	Population	Sampling strategy	Sampling method	Instrument criteria	Diagnostic criteria	Mean age (years)	Age range	Psychiatric interviewer^a^	Mean duration in prison	Type of prisoner	% male	No. committed violent offences	No. not consenting
Collins 1988	USA	North Carolina prisons	All males admitted March–June 1983	Consecutive new arrivals at reception	DIS^b^	DSM‐III^c^	27.6	Not reported	N	Not reported	Sentenced	100%	157	117
Daniel 1988	USA	Missouri Correctional Classification Center	Consecutive arrivals over 7 months	Consecutive sampling at reception	DIS	DSM‐III	29	SD 8.2	N	Not reported	Sentenced	0%	21	0
Teplin 1994	USA	Cook County Departmentof Corrections, Chicago	All remands 1983–84	Stratified randomsampling	DIS	DSM‐III‐R	Not reported	Not reported	Not reported	Not reported	Remand	100%	Not reported	35
Jordan 1996	USA	Correctional Institution for Women, Raleigh, NC	All sentenced incoming prisoners in 1991–92	Combined consecutive and random sampling	CIDI^d^	DSM‐III‐R	31.5	18–65	Y	5–10 days	Sentenced	0%	98	42
Smith 1996	Ireland	Mountjoy Prison, Dublin	All new arrivals in 1992–93	Simple random sampling	Clinical interview	DSM‐III‐R	Not reported	Not reported	Y	1 day	Mixed	100%	Not reported	2
Teplin 1996	USA	Cook County Departmentof Corrections, Chicago	All remands 1991–93	Stratified randomsampling	DIS	DSM‐III‐R	28	17–67	N	Not reported	Remand	0%	201	59
Mason 1997	England	Durham Remand prison for men	All remands over 7 months	Consecutive sampling at reception	Clinical interview	DSM‐IV	Not reported	Not reported	Y	Not reported	Remand	100%	Not reported	0
McClellan 1997	USA	Prison unit for men and reception centre for women, Texas	All newly admitted inmates	Simple random sampling	DIS	DSM‐III	32.8 male 32.3 female	Not reported	N	Not reported	Mixed	67%	Not reported	202
Mohan 1997	Ireland	Mountjoy Prison, Dublin	Consecutive new arrivals over 3 months	Simple random sampling	SCAN^e^	DSM‐IV	25.8	17–48	Y	Not reported	Mixed	0%	0	0
Peters 1998	USA	Holliday Transfer Facility, Texas	Consecutive new arrivals in 1996	Consecutive sampling at reception	SCID IV^f^	DSM‐IV	32.6	SD 10.2	Y	14–60 days	Sentenced	100%	61	100
Lo 2000	USA	Cuyahoga County Jail, Cleveland, USA	All sentenced incoming prisoners in 1997–98	Consecutive sampling	DIS	DSM‐IV	30	18–58	N	Not reported	Sentenced	76%	Not reported	29
Marquart 2001	USA	Texas Deptartment of Criminal Justice, institutional division	All female prisonersadmitted in 1994	Simple random sampling	DIS	DSM‐IV	32.3	17–63	Y	Not reported	Remand	0%	Not reported	0
Butler 2003	Australia	Metropolitan Remand and Reception Centre, female Correctional Centre and remote reception sites	Consecutive convenience sample of admissions over 3 months	Convenience sample among those admitted over 3 months	CIDI	DSM‐IV and ICD‐10^g^	Men 29.61, women 29.10	Not reported	Mental health nurses	Not reported	Mixed	100%	Not reported	Non‐screened: 67.4%
Wright 2006	Ireland	The Dochas Centre, female wing of Limerick Prison near Dublin	Consecutive admissions in August 2003 and between April 2004 and May 2004	All consenting prisoners interviewed at reception (10.7% of all committals)	SADS‐L,^h^ SODQ^i^	ICD‐10	27.4	Not reported	Post‐membership psychiatrists	Aimed to interview within 72 hours of reception	Mixed	0%	14/60 = 23.3%	30
Jones 2006	England	HMP Grendon (therapeutic community prison)	Consecutive admissions in 2003	All consenting prisoners interviewed at reception	CAAPE^j^	DSM‐IV	30.7	18–66	Psychological counsellor	Shortly after admission	Sentenced	100%	Not reported	0
Bulten 2009	Netherlands	Vught prison	Random sample of admissions to ‘general wards’ of prison	Random sample among new admissions	MINI^k^	DSM‐III‐R	30.4	18–59	Trained psychologist	First weeks of incarceration	Mixed	100%	73	50
Curtin 2009	Ireland	Cloverhill, Limerick and Cork Prisons (remand), Mountjoy and Cork Prisons (sentenced)	Consecutive admissions, up to 10 per day	All consenting prisoners interviewed at reception	SADS‐L	ICD‐10	29.8	18+	Post‐membership psychiatrists	Within 72 hours	Mixed	100%	79	54
Einarsson 2009	Iceland	Icelandic prison for sentenced inmates	All new admissions in study period (females excluded)	All consenting prisoners interviewed at reception	MINI 5	DSM‐IV	31	19–56	Psychologist	Within 10 days	Sentenced	100%	15	16
Stompe 2010	Austria	Prison Vienna‐Josefstadt	Consecutive recruitment of admissions	All eligible new admits.	SCAN	ICD‐10	Not reported	18+	Doctor (psychiatry trainee)	Not reported	Mixed	100%	Not reported	0
Proctor 2012	USA	Minnesota state prisons	All reception 2000–03	All consenting prisoners interviewed at reception	SUDDS‐IV^l^	DSM‐IV	32.8	18–58	Addictions counsellors (computer recorded interview)	Not reported	Sentenced	0%	Not reported	0
Sarlon 2012	France	Local prisons of Fleury‐Merogis, Loos, Lyon, Marseille	Reception: new receptions to local prisons in four areas	All consenting prisoners interviewed at reception	MINI plus 5.0	DSM‐IV	29.9	18–64	Clinicians (psychiatrist and psychologist)	within 14 days	Mixed	100%	Not reported	30
Tavares 2012	Brazil	Porto Alegre prison	Consecutive admissions	Random sample among new admits (calculation of 30 a base‐point for recruitment)	MINI‐plus (Brazilian version)	DSM‐IV	27.88	Not reported	Not reported	Within 3 months	Sentenced	100%	10	0
Mir 2015	Germany	Penal justice system in Berlin	Consecutive admissions screened for eligibility	All eligible new admits. Aimed for sample of 150.	MINI 6.0 (German version)	DSM‐IV	34.3	Not reported	Clinical psychologist	Within 1 month (usually <1 week)	Mixed	0%	0	48
Mundt 2015	Chile	Santiago Uno central facility, Centro Penitenciario Feminino, San Joaquín, CPF San Miguel central admission facilities	Consecutive admissions	All consenting prisoners interviewed at reception	MINI Spanish version	DSM‐IV	31.6	Not reported	Clinical psychologist/nurse (trained by senior consultant psychiatrist)	7.7 days	Remand	54%	127	30
Hoffmann 2015	USA	8 adult state prison facilities of Minnesota	Uses routine data collected on admissions, all admissions during 2002–03	All consenting prisoners interviewed at reception	SUDDS‐IV	ICD‐10	31	18–65	Addiction counsellors	On admission	Sentenced	90%	Not reported	0

a Y = Yes; psychiatrist, N = no; non‐psychiatrist (trained interviewer);

b DIS = Diagnostic Interview Schedule;

c DSM = Diagnostic and Statistical Manual of Mental Disorders; DSM‐IIIR = DSM‐III revised;

d CIDI = Composite International Diagnostic Interview;

e SCAN = Schedules for Clinical Assessment in Neuropsychiatry;

f SCID = Structured Clinical Interview for DSM Disorders;

g ICD = International Classification of Diseases;

h SADS‐L = Schedule for Affective Disorders and Schizophrenia – life‐time version;

i SODQ = Severity of Opiate Dependence Questionnaire;

j CAAPE = Comprehensive Addictions and Psychological Evaluation;

k MINI = Mini International Neuropsychiatric Interview;

l SUDDS = Substance Use Disorders Diagnostic Schedule.

**Table 2:** Prevalence estimates of substance use disorder in reception studies of prisoners.

Study	Total no.	Males (%)	No. with alcohol use disorder	No. with drug use disorder	Prevalence of alcohol use disorder (%)	Prevalence of drug use disorder (%)
Daniel 1988	100	0	10^a^	–	10.0	–
Collins 1988	1120	100	302^a^	112^a^	27.0	10.0
Teplin 1994	728	100	116^a^	129^a^	15.9	17.7
Jordan 1996	805	0	244^a^	138^a^	30.3	17.1
Smith 1996	235	100	63	46	26.8	19.6
Teplin 1996	1272	0	667^a^	304^a^	52.4	23.9
Bushnell 1997	100	100	19^a^	14^a^	19.0	14.0
Mason 1997	548	100	116^a^	214^a^	21.2	39.1
McClellan 1997	1030 male 500 female	67	309 male 93 female	331 male 227 female	30.0 male 18.6 female	32.1 male 45.4 female
Mohan 1997	45	0	0	26	0.0	57.8
Peters 1998	400	100	86^a^	100^a^	21.5	25.0
Lo 2000	152 male 48 female	76	–	73 male 29 female	–	48.0 male 60.4 female
Marquart 2001	500	0	88^a^	224^a^	17.6	44.8
Butler 2003	756 male 165 female	82	142 male 27 female	378 male 111 female	19.2 male 16.5 female	52.0 male 68.9 female
Wright 2006	94	0	23	45	24.7	48.4
Jones 2006	118	100	53	–	44.9	–
Bulten 2009	191	100	53	57	27.7	29.8
Curtin 2009	615	100	148	206	24.1	33.5
Einarsson 2009	90	100	46	55	51.1	61.1
Stompe 2010	200	100	59^a^	–	29.5	–
Proctor 2012	801	0	242	456	30.2	56.9
Sarlon 2012	267	100	43	47	16.1	17.6
Mir 2015	150	0	31	71	20.7	47.3
Hoffmann 2015	6871	90	2177	–	31.7	–
LMI countries						
Tavares 2012	60	100	26	18	43.3	30.0
Mundt 2015	229 male 198 female	54	68 male 23 female	128 male 47 female	29.7 male 11.6 female	55.9 male 23.7 female
Juvenile prisoners						
Köhler 2009	149	100	31	–	20.8	–
Vreugdenhil 2003	204	100	45	–	22.1	–
McClelland 2004	1143 male 631 female	64	289 male^a^ 156 female^a^	276 male^a^ 260 female^a^	25.3 male 24.7 female	24.1 male 41.2 female
Plattner 2012	275	100	45	135	16.4	49.1
Dixon 2005	100	0	55^a^	85^a^	55.0	85.0

a Figures for combined abuse and dependence; the rest are dependence only.

### Alcohol use disorder

**Figure 2:**
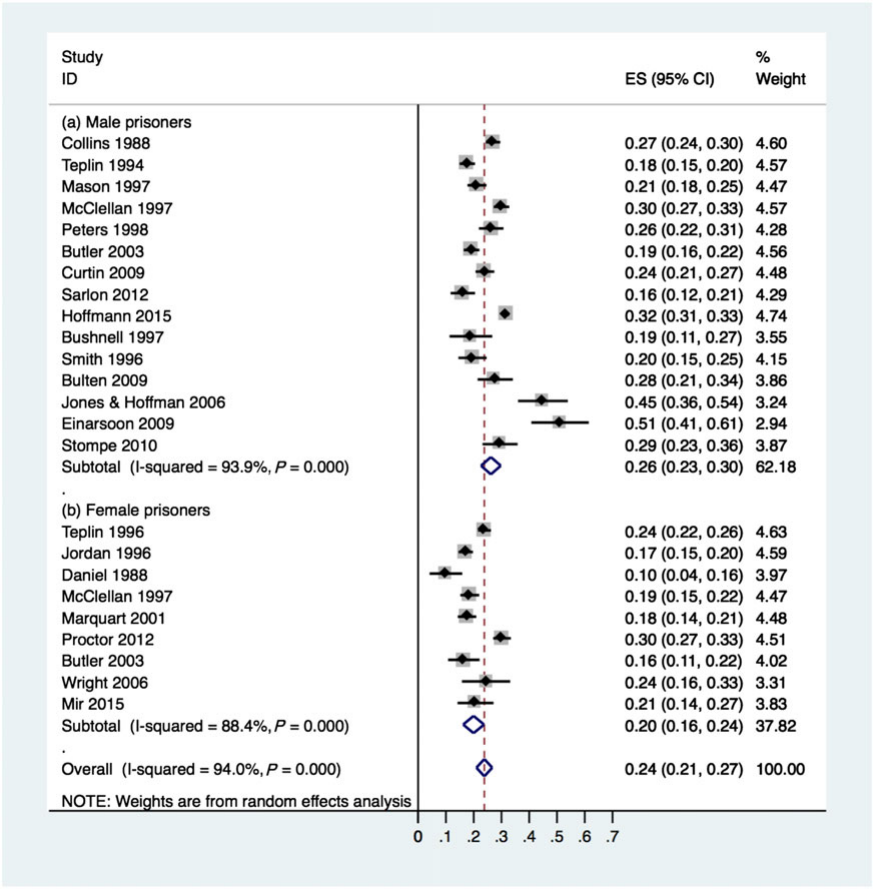
Prevalence of alcohol use disorder in male and female prisoners on reception to prison (ES = prevalence estimates). [Colour figure can be viewed at wileyonlinelibrary.com]

The overall pooled prevalence estimate of alcohol use disorder was 24% [95% confidence interval (CI) = 21–27], with very high levels of between‐study heterogeneity (*I* ^2^ = 94%; 95% CI = 92–95). Fifteen studies of alcohol use disorder in men were identified in 12 739 prisoners 37, 38, 40, 41, 42, 44, 48, 50, 51, 52, 57, 58, 60, 61. Pooled prevalence estimate for males was 26% (95% CI = 23–30), with substantial heterogeneity between studies (*I* ^2^ = 94%; 95% CI = 92–96) and a range of 16–51% in individual studies. We identified 10 investigations that measured alcohol use disorder in female prisoners 38, 39, 40, 45, 46, 49, 53, 54, 55, 56, and pooled prevalence estimate was 20% (95% CI = 16–24) with high heterogeneity (*I* ^2^ = 88%; 95% CI = 80–93). Primary studies provided estimates that varied from 10 to 30% (Figure 2).

Two investigations in LMI countries reported prevalences of 43% 25 and 30% 26. There were four investigations of alcohol use disorder in juvenile men, and prevalences ranged from 16 to 25% 27, 28, 29, 31).

### Drug use disorder

There was evidence of heterogeneity by sex in univariable meta‐regression, and prevalence estimates for drug use disorder are stratified accordingly.

#### Men

Thirteen studies reported drug use disorder in male prisoners 37, 38, 40, 41, 42, 44, 47, 48, 50, 51, 52, 57, 60. The pooled prevalence estimate was 30% (95% CI = 22–38), with very high heterogeneity (*I* ^2^ = 98%; 95% CI = 98–99). These varied from 10 to 61%. In LMI countries, reported prevalences were 30% 25 and 56% 26.

#### Women

Ten relevant studies on drug use disorder in female prisoners were identified 38, 39, 40, 46, 47, 49, 53, 54, 55, 56. The pooled prevalence estimate was 51% (95% CI = 43–58) with substantial heterogeneity (*I* ^2^ = 95%; 95% CI = 93–97). Prevalences ranged from 30 to 69%.

### Sources of heterogeneity

In univariable meta‐regression (*n* = 23 studies), factors associated with heterogeneity included: females reported higher drug use disorder than males (β = 0.21; 95% CI = 0.33–0.10; *P* = 0.001), more recent studies (published after 2000) reported higher rates of drug use disorder (β = 0.15; 95% CI = 0.12–0.28; *P* = 0.03), and participation rate was associated negatively with drug use disorder (β = −0.37; 95% CI = 0.73, −0.01; *P* = 0.045). No significant associations were reported with alcohol use disorder, although there was a non‐significant link with publication year as a continuous variable (β = 0.004; 95% CI = –0.00002, 0.008; *P* = 0.051).

**Figure 3:**
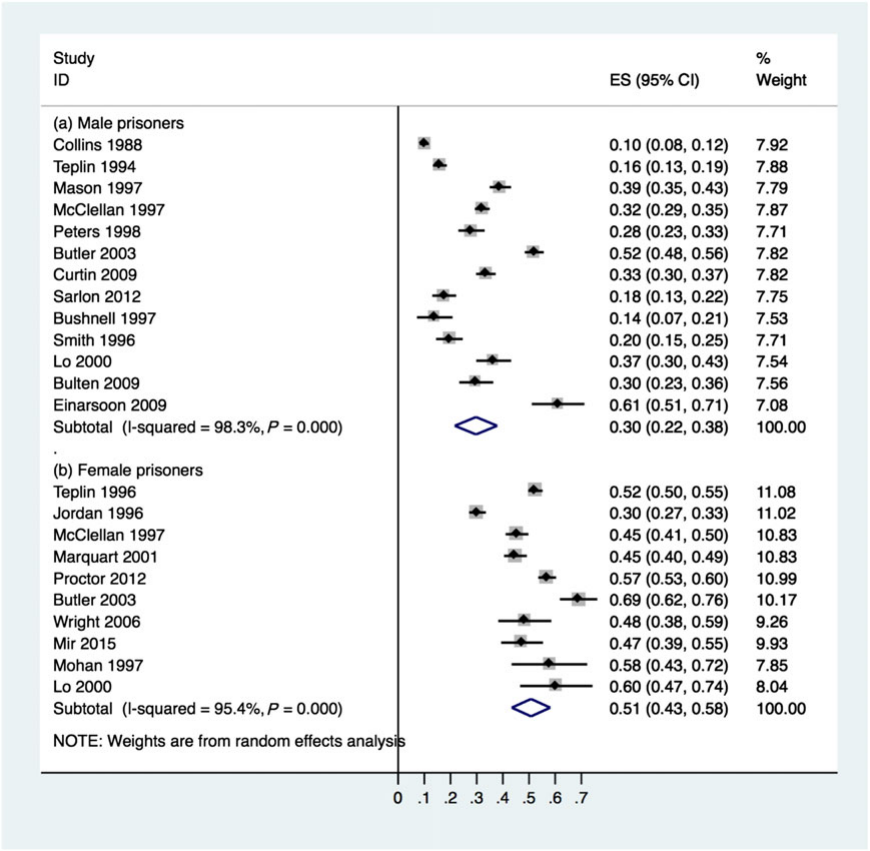
Prevalence of drug use disorder in male and female prisoners on reception to prison (ES = prevalence estimates). [Colour figure can be viewed at wileyonlinelibrary.com]

Using subgroup analysis, we also investigated possible explanations for between‐study variation (Table 3). This found that there were higher estimates for drug use disorders in women, and for both drug and alcohol use disorders since 2000, which were consistent with findings on meta‐regression. In addition, in alcohol use disorders, there were higher prevalence estimates in sentenced (than remand) prisoners. However, these subgroup analyses had overlapping CIs, apart from a higher estimate for women with drug use disorder compared to men (Figure 3).

**Table 3:** Pooled prevalence estimates for drug and alcohol use disorders in newly incarcerated men and women by pre‐specified subgroups.

	Alcohol use disorder, % (95% CI)		Drug use disorder, % (95% CI)	
	Male	Female	Male	Female
Country				
High income countries	–	–	30 (22–38) (*n* = 5750; *k* = 13)	51 (43–58) (*n* = 4379; *k* = 10)
USA	23 (19–27) (*n* = 9619; *k* = 5)	20 (15–25) (*n* = 3978; *k* = 6)	37 (26–48)(*n* = 2948; *k* = 5)	48 (39–57) (*n* = 3926; *k* = 6)
Non‐USA	25 (21–28) (*n* = 3573; *k* = 14)	20 (15–24) (*n* = 453; *k* = 4)	40 (31–50) (*n* = 3255; *k* = 12)	56 (44–68) (*n* = 453; *k* = 4)
Publication year				
Before 2000	–	–	–	46 (33–58) (*n* = 2622; *k* = 4)
2000 and after	–	–	–	54 (47–62) (*n* = 1757; *k* = 6)
Prisoner type				
Remand	21 (18–25) (*n* = 1502; *k* = 4)	–	–	–
Sentenced	33 (29–37) (*n* = 8808; *k* = 7)	–	–	–
Interviewer				
Psychiatrist	23 (19–26) (*n* = 2265; *k* = 6)	–	–	–
Other	30 (26–35) (*n* = 9746; *k* = 8)	–	–	–

CI = confidence interval.

#### Publication bias

There was no evidence of publication bias overall and in subgroups stratified by sex apart from drug use disorder in male prisoners, where there was non‐significant evidence of publication bias in the funnel plot analysis (Egger's test, *t* = 2.19, SE(*t*) = 4.27, *P* = 0.051) 37, 38, 40, 41, 42, 44, 47, 48, 50, 51, 52, 57, 60. Visual analysis of the funnel plot suggested asymmetry, but appeared to be mainly attributable to one study 60 with a high prevalence and large standard error, which when removed did not suggest clear publication bias (Supporting information, Appendix S4).

## Discussion

This updated systematic review of the prevalence of substance use disorder in prisoners is based on 24 studies and 18 388 individuals in 10 countries. In addition, we identified five studies in juvenile prisoners and two investigations in LMI countries. The sample size in this update is more than double of that a previous systematic review 11, which identified relevant prevalence studies until 2004, and this updated synthesis allowed for an investigation of sources of heterogeneity between included studies.

We report two main findings. The first is that alcohol use disorder was highly prevalent in prisoners, with a pooled estimate of 24% (95% CI = 21–27). In men, the lowest estimate suggests that one in six (16%) met the threshold for alcohol use disorder on arrival into prison, and in women it was one in 10. By way of comparison, in the United States in 2013 community rates of past year alcohol use disorder were estimated at 8.7% for men and 4.6% in women 62. According to the Global Burden of Disease 2015 Study, the global prevalence of alcohol use disorder was 1.5% for males and 0.3% for females (0.9% for both sexes) 63. The second major finding was that drug use disorder was as high as the alcohol estimates, and possibly higher in female prisoners, with a pooled estimate of 51% (95% CI = 43–58). Importantly, the lowest prevalence study in women found that 30% had a drug use disorder. This can be contrasted with US community samples, where 3.4% of men and 1.9% women had such a disorder 62, and 0.8% in men and 0.4% in women (0.6% for both sexes) worldwide 63.

We investigated sources of heterogeneity more carefully than previous work, which led to a number of potentially important findings. First, using meta‐regression, we found evidence of increasing drug use disorder in prison studies during the past three decades. This is in contrast with community trends in some high‐income countries such as the United States, where drug use disorder had not increased (and alcohol reduced slightly) between 2000 and 2013 64. Secondly, two other study characteristics were associated with significant variations in prevalence. Having a higher participation rate was associated with lower rates in drug use disorder, and there were higher rates of drug use disorders in women prisoners. Being assessed by a psychiatrist was also linked with lower alcohol use disorder prevalence in subgroup analyses, although the confidence intervals overlapped. This should inform the interpretation of single studies, particularly if used for service planning and development. One possible explanation for heterogeneity that we did not investigate are the community baseline rates of substance use disorders, and future work could examine this using comparable measures of drug and alcohol use, such as the ongoing Global Burden of Disease 63. In addition, the reported high prevalence range of 30–56% for substance use disorder in LMI countries needs further research, as it was based on only two investigations.

A number of implications arise from this updated meta‐analysis. First, it highlights the opportunity that jails and prisons present to treat substance use disorders 65. The high prevalences underscore the importance of evidence‐based interventions being available to all individuals entering custody. Four areas should be considered to improve management of substance use disorders in prisoners. First, prison arrival centres need to have systems in place to identify individuals with high treatment needs, and treatments should be matched to individual needs 65. Secondly, acute detoxification management should be available to all entrants to custody, which may include short‐term prescription of benzodiazepines for alcohol withdrawal 66 and symptomatic treatment of withdrawal from other substances that may include opioid agonists (such as methadone or buprenorphine). Detoxification programmes may benefit from the use of clinical tools to document withdrawal symptoms 67. Thirdly, combination pharmacological and psychosocial treatments should be available, considering the high prevalences and the subsequent effects on adverse outcomes, including mortality after release and violent re‐offending 68, 69. Finally, considering the high relapse rates, programmes need to link prisoners with community services. Structured, simple and scalable tools to identify those at highest risk 70 and case management 71 may assist in this process. A second implication from the review is that prevalence research needs to consider some areas of improvement. These include separating prevalences by drug and alcohol use disorder, and also providing information stratified by sex and prisoner status (i.e. sentenced or not). Baseline information on socio‐demographic and criminal history characteristics (such as those listed in Table 1, including the sample's age structure and index offence) should be provided in new studies, and supplemented with more clinically informative information, such as comorbidities with mental illness 72 and chronic pain, prevalence by individual drugs and most recent treatment. At the same time, as there are now at least 24 studies on prevalence on more than 18 000 prisoners, whether new research should prioritize how treatment can be delivered most effectively to prisoners and former prisoners needs to be considered by funding agencies, researchers and government agencies in criminal justice and public health.

Some limitations to this review need to be considered. First, there was variation in the diagnostic tools and interviewers used to assess substance use disorders, and we found that psychiatrist interviewers were associated with lower prevalences for alcohol use disorder. To reflect this clinical and statistical heterogeneity, we also reported prevalence ranges. Secondly, as we focused upon substance use disorders on prison entry, these estimates may not reflect treatment needs later in prison or on prison release, where novel psychoactive substances are increasingly problematic and may require different treatment approaches 73. In addition, the misuse of prescribed medication such as painkillers, anti‐epileptics and anxiolytics inside custody needs to be considered, and may further increase treatment needs. Finally, some of the subgroup analyses were based on fewer than 10 studies, and should be interpreted with caution.

In summary, the high prevalence of alcohol and drug use disorders in prisoners remains a key challenge for prison health. Tackling this will probably require interventions at all stages of the criminal justice process—from identifying and treating withdrawal in police custody 74 and on arrival to prison, to opiate maintenance and other treatments during any period in prison 68, to community links being made and integrated treatment provided on release 75. Comprehensive strategies to prevent relapse of substance dependence are likely to reduce premature mortality, recidivism and subsequent return to prison.

### Declaration of interests

None.

## Supporting information


**Appendix S1** Preferred Reporting Items for Systematic Reviews and Meta‐Analyses (PRISMA) checklist.
**Appendix S2** Quality checklist.
**Appendix S3** Study characteristics of studies of substance use disorders in juvenile prisoners.
**Appendix S4** Funnel plot of studies reporting drug use disorder prevalence in male prisoners.
