# Supplementary material for: Substance use disorders in prisoners: an updated systematic review and meta‐regression analysis in recently incarcerated men and women
Source: Addiction. 2017 Jun 28;112(10):1725–39. doi: 10.1111/add.13877 (PMC5589068; doi:10.1111/add.13877)

**Appendix A.** PRISMA Checklist

| **Section/topic** | **#** | **Checklist item** | **Reported on page #** |
| --- | --- | --- | --- |
| **TITLE** | | |  |
| Title | 1 | Identify the report as a systematic review, meta-analysis, or both. | 1 |
| **ABSTRACT** | | |  |
| Structured summary | 2 | Provide a structured summary including, as applicable: background; objectives; data sources; study eligibility criteria, participants, and interventions; study appraisal and synthesis methods; results; limitations; conclusions and implications of key findings; systematic review registration number. | 3 |
| **INTRODUCTION** | | |  |
| Rationale | 3 | Describe the rationale for the review in the context of what is already known. | 4 |
| Objectives | 4 | Provide an explicit statement of questions being addressed with reference to participants, interventions, comparisons, outcomes, and study design (PICOS). | 3-4 |
| **METHODS** | | |  |
| Protocol and registration | 5 | Indicate if a review protocol exists, if and where it can be accessed (e.g., Web address), and, if available, provide registration information including registration number. | 5 |
| Eligibility criteria | 6 | Specify study characteristics (e.g., PICOS, length of follow-up) and report characteristics (e.g., years considered, language, publication status) used as criteria for eligibility, giving rationale. | 5-6 |
| Information sources | 7 | Describe all information sources (e.g., databases with dates of coverage, contact with study authors to identify additional studies) in the search and date last searched. | 4-5 |
| Search | 8 | Present full electronic search strategy for at least one database, including any limits used, such that it could be repeated. | 5 |
| Study selection | 9 | State the process for selecting studies (i.e., screening, eligibility, included in systematic review, and, if applicable, included in the meta-analysis). | 5-6 |
| Data collection process | 10 | Describe method of data extraction from reports (e.g., piloted forms, independently, in duplicate) and any processes for obtaining and confirming data from investigators. | 6-7 |
| Data items | 11 | List and define all variables for which data were sought (e.g., PICOS, funding sources) and any assumptions and simplifications made. | 6-7 |
| Risk of bias in individual studies | 12 | Describe methods used for assessing risk of bias of individual studies (including specification of whether this was done at the study or outcome level), and how this information is to be used in any data synthesis. | 7 |
| Summary measures | 13 | State the principal summary measures (e.g., risk ratio, difference in means). | 6-7 |
| Synthesis of results | 14 | Describe the methods of handling data and combining results of studies, if done, including measures of consistency (e.g., I^2^) for each meta-analysis. | 6-7 |
| Risk of bias across studies | 15 | Specify any assessment of risk of bias that may affect the cumulative evidence (e.g., publication bias, selective reporting within studies). | 7 |
| Additional analyses | 16 | Describe methods of additional analyses (e.g., sensitivity or subgroup analyses, meta-regression), if done, indicating which were pre-specified. | 6-7 |
| **RESULTS** | | |  |
| Study selection | 17 | Give numbers of studies screened, assessed for eligibility, and included in the review, with reasons for exclusions at each stage, ideally with a flow diagram. | 7, 18 |
| Study characteristics | 18 | For each study, present characteristics for which data were extracted (e.g., study size, PICOS, follow-up period) and provide the citations. | 7-8 |
| Risk of bias within studies | 19 | Present data on risk of bias of each study and, if available, any outcome level assessment (see item 12). | 10 |
| Results of individual studies | 20 | For all outcomes considered (benefits or harms), present, for each study: (a) simple summary data for each intervention group (b) effect estimates and confidence intervals, ideally with a forest plot. | 8-9 |
| Synthesis of results | 21 | Present results of each meta-analysis done, including confidence intervals and measures of consistency. | 8-9 |
| Risk of bias across studies | 22 | Present results of any assessment of risk of bias across studies (see Item 15). | 10 |
| Additional analysis | 23 | Give results of additional analyses, if done (e.g., sensitivity or subgroup analyses, meta-regression [see Item 16]). | 9-10 |
| **DISCUSSION** | | |  |
| Summary of evidence | 24 | Summarize the main findings including the strength of evidence for each main outcome; consider their relevance to key groups (e.g., healthcare providers, users, and policy makers). | 11-12 |
| Limitations | 25 | Discuss limitations at study and outcome level (e.g., risk of bias), and at review-level (e.g., incomplete retrieval of identified research, reporting bias). | 11 |
| Conclusions | 26 | Provide a general interpretation of the results in the context of other evidence, and implications for future research. | 12 |
| **FUNDING** | | |  |
| Funding | 27 | Describe sources of funding for the systematic review and other support (e.g., supply of data); role of funders for the systematic review. | 2 |

**Appendix B.** Quality checklist

**JBI Critical Appraisal Checklist for Studies Reporting Prevalence Data**

Reviewer



Date

Author



Year



Record Number


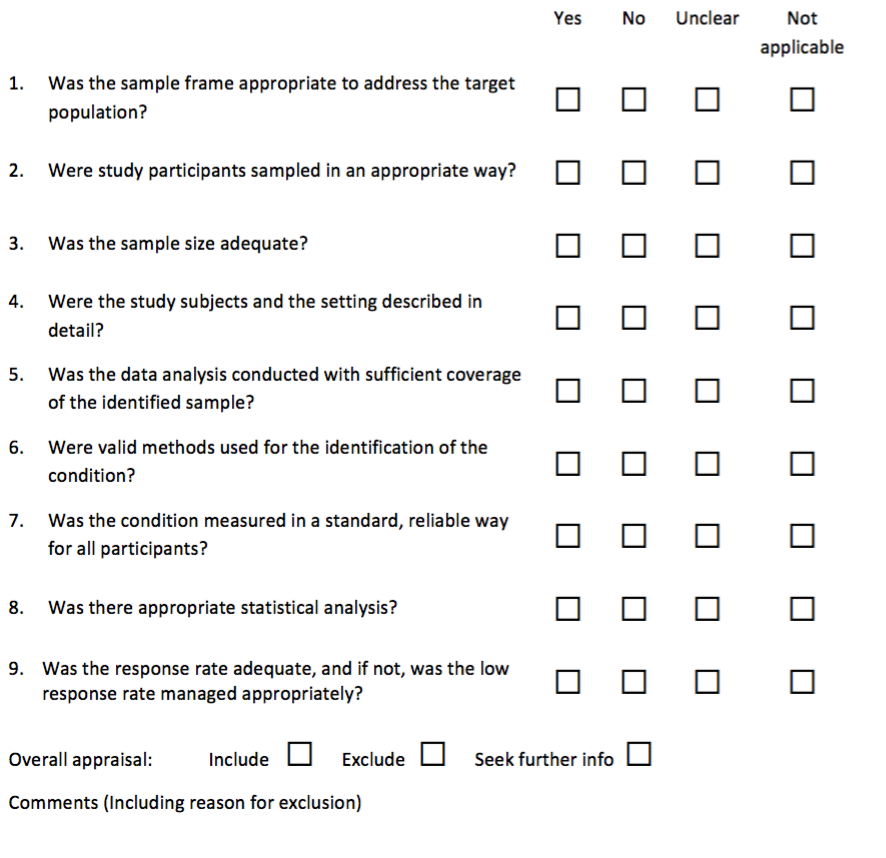


**Appendix C.** Study characteristics of studies of substance use disorders in juvenile prisoners.

| **Study** | **Country** | **Population** | **Sampling strategy** | **Sampling method** | **Instrument criteria** | **Diagnostic criteria** | **Mean age (years)** | **Age Range** | **Psychiatric interviewer** | **Mean duration in prison** | **Type of prisoner** | **% male** | **No committed violent offences** | **No not consenting** |
| --- | --- | --- | --- | --- | --- | --- | --- | --- | --- | --- | --- | --- | --- | --- |
| Vreugdenhil 2003 | Netherlands | 6 of 9 youth detention centres in Netherlands | Consecutive sample (cross-sectional for demographics) | All consenting prisoners interviewed at reception | DISC 2.3 Dutch version | DSM-III-R | 16.4 | Not reported | Clinical psychologists | Not reported | Sentenced | 100% | 0.58 | 82 |
| McClelland 2004 | USA | Cook County Juvenile Temporary Detention Center | Sample on admission | Random sample on admission, stratified by sex, ethnicity, age and legal status | DISC 2.3 | DSM-III-R | 14.9 | 10 to 18 | Trained interviewers (non-clinicians) | Within 2 days of intake | Sentenced | 64% | Not reported | 96 |
| Dixon 2005 | Australia | Female state juvenile prison in Sydney | A sample of admissions over 15 months. | Random sample among new admissions. Excluded those with physical volatility | K-SADS-PL | DSM-IV | 16.5 | 13.5-19 | Clinical psychologist | First 2 months of detention | Sentenced | 0% | 0.71 | 5 |
| Köhler 2009 | Germany | Juvenile prison of Neumünste | Consecutive recruitment of admissions during 15 month period (01-03). | All eligible new admits. | SCID | DSM-IV | 19.07 | 15-25 | Not reported (structured clinical interview) | 8.75 days | Mixed | 100% | 22.1% | 12 |
| Plattner 2012 | Switzerland | Vienna County Jail (male juvenile remand) | All admissions between 2003 and 2005. | All eligible new admits. | MINI-KID (A German version) | DSM-IV and ICD-10 | 16.45 | 14-20 | Child and adolescent psychiatry residents | Within 4 days | Remand | 100% | 181 | 8 |

**Appendix D.** Funnel plot of studies reporting drug use disorder prevalence in male prisoners.


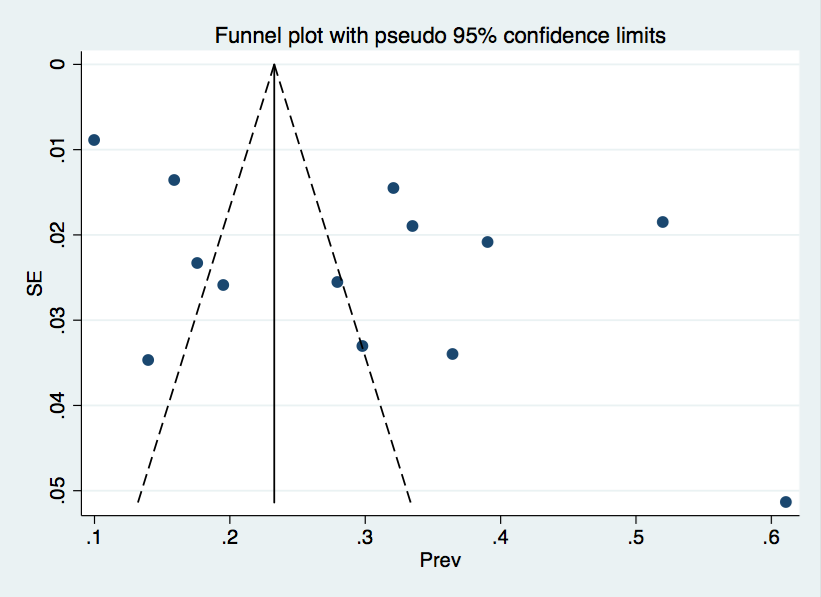

Supplement: Supplementary file 1 — Appendix S1 Preferred Reporting Items for Systematic Reviews and Meta‐Analyses (PRISMA) checklist. Appendix S2 Quality checklist. Appendix S3 Study characteristics of studies of substance use disorders in juvenile prisoners. Appendix S4 Funnel plot of studies reporting drug use disorder prevalence in male prisoners. [file ADD-112-1725-s001.docx]
